# Paroxysmal Sympathetic Hyperactivity in Moderate-to-Severe Traumatic Brain Injury and the Role of Beta-Blockers: A Scoping Review

**DOI:** 10.1155/2021/5589239

**Published:** 2021-09-11

**Authors:** Stéphane Nguembu, Marco Meloni, Geneviève Endalle, Hugues Dokponou, Olaoluwa Ezekiel Dada, Wah Praise Senyuy, Ulrick Sidney Kanmounye

**Affiliations:** ^1^Research Department, Association of Future African Neurosurgeons, Yaounde, Cameroon; ^2^Higher Institute of Health Sciences, Université des Montagnes, Bangangté, Cameroon; ^3^Faculty of Health Sciences, University of Buea, Buea, Cameroon; ^4^Department of Medicine and Surgery, Faculty of Clinical Sciences, College of Medicine, University of Ibadan, Ibadan, Oyo State, Nigeria

## Abstract

**Introduction:**

Most cases of paroxysmal sympathetic hyperactivity (PSH) result from traumatic brain injury (TBI). Little is known about its pathophysiology and treatment, and several neuroprotective drugs are used including beta-blockers. The aim of our study is to collate existing evidence of the role of beta-blockers in the treatment of PSH.

**Methods:**

We searched MEDLINE, ResearchGate, and Google Scholar, for keywords related to PSH and the role of beta-blockers in moderate-to-severe TBI on September 23, 2020. Two authors blindly screened the articles found with Rayyan. Both resolved their conflicts by mutual consent. If no solution was found, a third author was consulted. Simple descriptive data analysis was performed and the results were presented both in a narrative and tabular form.

**Results:**

Of the 19 items found, 10 met the criteria for inclusion. 50% were systematic reviews without meta-analysis, 40% were observational studies, and 10% were experimental studies. Propranolol was the main beta-blocker found in 80% of the studies and was the only molecule used in the treatment of paroxysmal sympathetic hyperactivity in 40% of the included studies. Only two studies evaluated and showed a significant association between beta-blockers and mortality rate (5.1% vs. 10.8%; *P*=0.03), (3% vs. 15%; *P*=0.002), respectively.

**Conclusion:**

Propranolol is the beta-blocker that has been shown to be effective in reducing the length of stay and mortality rate in moderate-severe traumatic brain injury patients with PSH. However, further studies are needed to precisely define the terms and conditions of its use.

## 1. Introduction

In 1929, Wilder Penfield described a syndrome combining lacrimation, hypertension, diaphoresis, and agitation. He named this syndrome a diencephalic seizure [1]. Electrophysiological investigations of this phenomenon did not show electroencephalographic activity. Many names were attributed to this syndrome: dysautonomia, sympathetic storming, brainstem attack, autonomic dysregulation, and paroxysmal autonomic instability with dystonia [2–5]. In 2014, the International Brain Injury Association convened a consensus workgroup to clarify its nomenclature and diagnostic criteria. The proposed term from this consensus group was “paroxysmal sympathetic hyperactivity (PSH)” [6].

PSH traditionally occurs in severe acquired injuries such as traumatic brain injury (TBI), stroke, anoxic brain injury, tumors, infections, spinal injuries, and serotonin syndrome. The prevalence of PSH is 8–33% [7] and 79.4% of PSH is due to TBI [8]. 80% of PSH patients have moderate-to-severe TBI and 15–33% of severe TBI patients have PSH [7].

The pathophysiology of PSH is poorly understood and the dominant theory suggests the failure of the central autonomic network (insular cortex, amygdala, hypothalamus, medulla, periaqueductal gray matter, parabrachial complex, and nucleus of the tractus solitarius) [9, 10].

PSH has several symptoms variably present: they are well resumed by the consensus position, which defines PSH as a “syndrome of simultaneous, paroxysmal transient increased in sympathetic (elevated heart rate, blood pressure, respiratory rate, temperature, sweating) and motor (posturing) activity” [6].

Many pharmacological agents have been used alone or in combination to treat PSH. They include opiates, dopamine agonists, benzodiazepines, baclofen, gabapentin, and beta-blockers [9]. Beta-blockers have a cardioprotective effect: reducing the heart rate, perfusion volume, and mean arterial pressure. This effect limits myocardial oxygen consumption, thus preventing myocardial infarction. Also, beta-blockers have a neuroprotective effect by reducing cerebral blood flow, which reduces the cerebral consumption of oxygen and glucose as metabolism is reduced. Nonselective beta receptor antagonists have a lipophilic property that allows them to cross the blood-brain barrier. As a result, beta-blockers diminish the effect of circulating catecholamines and attenuate the resting metabolism rate, which is markedly increased in patients with severe acute brain injury [11–18]. Propranolol is commonly used to treat PSH and labetalol has shown some effect against PSH [19, 20].

The aim of our review is to collate existing evidence of the role of beta-blockers in the treatment of PSH.

## 2. Method

### 2.1. Literature Search Strategy

The first author (SN) developed a comprehensive search strategy which was tested and adjusted by means of a pilot search. The authors searched PubMed and Google Scholar databases for keywords related to PSH and the role of beta-blockers in moderate-to-severe TBI on September 23, 2020. The search strategy included title, abstract, text word, and controlled vocabulary terms for PSH and beta-blockers. All databases were searched from their inception without language and date restrictions. Two authors (SN and USK) performed supplemental hand searches to identify additional publications. The complete research strategies and research terms are available in Appendix 1.

### 2.2. Data Extraction and Selection Criteria

Results were exported to a free online screening system, Rayyan QCRI (Doha, Qatar). Records were deduplicated and independently reviewed by four authors (MM, EG, DO, and HD). Included articles had to meet the following criteria:

1. Studies describing PSH secondary to TBI
2. Studies describing the treatment of PSH with beta-blockers alone or in combination

Conflicts were resolved through discussion and consensus between the two author reviewers and when this was not possible, a fifth reviewer (USK) was consulted.

Authors, study subject, study design, publication date, TBI severity, beta-blockers used and dosage, length of stay, and mortality were extracted from included studies. The data extracted were input into a Microsoft Excel spreadsheet (Microsoft, WA, USA), and the results were presented in a Table 1.

## 3. Results

**Figure 1:**
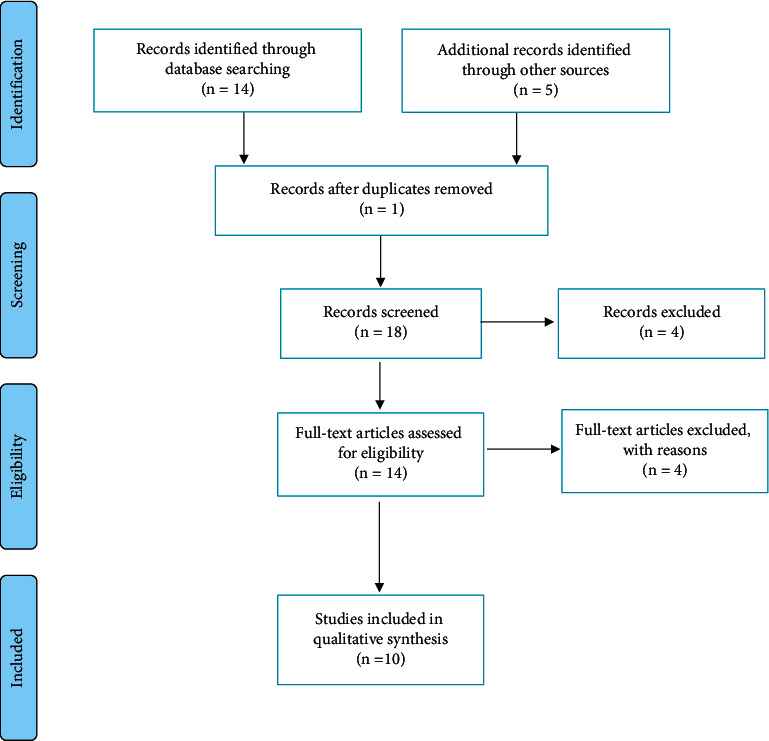
PRISMA flow diagram.

The search returned 19 articles: 14 (73.7%) through the systematic search of databases and 5 (26.3%) through the supplementary hand search. A duplicated article was excluded and the titles and abstracts of the 18 (94.7%) remaining articles were screened. Four (21.1%) did not match the inclusion criteria. Full-text review of the 14 (73.7%) remaining articles was done. Four (21.1%) articles were excluded because they did not involve moderate-to-severe TBI. Ten (52.6%) articles were included in the final data extraction and quantitative analysis (Figure 1).

Among the ten articles included in this review, five (50.0%) were reviews, 4 (40.0%) were observational studies, and one (10.0%) was an experimental study. Among the observational studies, 3 were case reports and 1 was a case-control study. The experimental study was a randomized, double-blinded, placebo-controlled trial.

Five (50.0%) studies had severe TBI patients, propranolol was used in 80.0% of the studies, and it was administered either intravenously (between 6 and 85 mg per 24 hours) or orally (20–60 mg every 4–6 hours). Other beta-blockers included labetalol (20.0%, *n* = 2 studies), atenolol (10.0%, *n* = 1 study), and metoprolol (10.0%, *n* = 1 study). These drugs were used in combination with propranolol and labetalol.

Beta-blockers were used in combination with non-beta-blocker drugs in five studies (50.0%.) The non-beta-blocker drugs included *α*2-agonists, gabapentin, baclofen, bromocriptine, long-acting benzodiazepines, dantrolene, morphine, and fentanyl.

Only two studies (20.0%) established the efficacy of beta-blockers on moderate-to-severe TBI patients with PSH (Table 1).

van der Jagt and Miranda [25] found that the mortality of PSH following moderate-to-severe TBI treated with beta-blockers was lower than for patients who did not receive beta-blockers (5.1% vs. 10.8% *P*=0.03).

Of note, Schroeppel et al. [24] found that patients who received beta-blockers had a longer hospital stay and higher mortality than patients who did not receive beta-blockers (29 days vs. 10 days and 13% vs. 6%, respectively, *P*=0.001). Among the patients who received beta-blockers, those who received propranolol had a longer stay, but mortality was significantly low. (44 days vs. 29; *P*=0.001) days, mortality was significantly lower (3% vs. 15%; *P*=0.002).

## 4. Discussion

### 4.1. Summary of Evidence

The role of beta-blockers in the management of PSH following moderate-to-severe TBI is a fairly recent concept. Before 2010, there was a single study [21], but nine studies have since been published [7, 9, 11, 22–27]. The studies were mostly systematic reviews without meta-analysis (40%) [7, 9, 22, 25] and case reports (30%) [21, 26, 27]. Only two studies evaluated the impact of beta-blockers on post-TBI PSH-related mortality, showing that beta-blocker therapy improved mortality [25]. One study showed a shortened length of stay [27]. However, the randomized controlled trial did not confirm the causal link between beta-blocker therapy and reduced mortality or length of stay [11]. Of note, beta-blockers were often used in combination with other drugs to manage post-TBI PSH (Table 1) [7, 9, 21–23].

Schroeppel et al. [24] performed a case-control study evaluating the use of beta-blockers in severe TBI, comparing propranolol to other beta-blockers. They found that the intervention group (i.e., propanol) had significantly longer lengths of stay (*P*=0.001) but significantly lower mortality rates (*P*=0.002). However, we note that the intervention group in this study was significantly younger. It can be argued that this group suffered from true PSH. PSH can be diagnosed early clinically [28–31] in the presence of > one episode of four or more of the following features: (1) fever (body temperature >38.3°C), (2) tachycardia (heart rate >120 beats per min in the absence of beta-blocker therapy or >100 beats per min during beta-blocker therapy), (3) hypertension, (systolic blood pressure >160 mmHg in the absence of beta-blocker therapy or >140 mmHg during beta-blocker therapy), (4) tachypnea (respiratory rate >25 breaths per min), (5) diaphoresis, (6) change in or presence of posturing, and (7) spasticity or rigidity. PSH is suspected if these symptoms cannot be explained by alternative causes including airway obstruction, sepsis, drug (e.g., neuroleptic malignant syndrome or serotonin syndrome), pulmonary embolism, withdrawal symptoms, or new or worsening structural brain injury.

PSH can be a devastating condition. PSH patients have higher mortality rates (OR = 0.08; 95% CI = 0.01–0.52) and tend to be older than other TBI patients (OR = 1.08; 95% CI = 1.00–1.16) [32]. In a retrospective study, Cotton et al. [25] reported lower mortality rates with beta-blocker therapy despite older age, higher injury severity scores, and lower estimated probability of survival (5.1% vs. 10.8%, *P*=0.03). Patients who received beta-blockers equally had more infectious complications, respiratory failure, and longer lengths of stay. In PSH patients, these complications are compounded by an increased likelihood of metabolic disorders, dehydration, and malnutrition [33]. Each of these comorbidities can adversely affect the mortality rate in patients with moderate-to-severe TBI. Therefore, these patients should be monitored closely [34–37].

We did not find consensus data on the time frame of administration, dose, criteria for withdrawal, and the long-term effects (e.g., quality of life measures) of beta-blocker therapy in moderate-to-severe TBI patients with PSH. These gaps in the existing literature warrant further studies.

### 4.2. Limitations

The limitations of our study are intrinsic to its typology (scoping review): its choice was dictated by the elements of heterogeneity of each article in the final data extraction (combination with several beta-blockers, different dosage, and withdrawal of beta-blockers, lack of data concerning length of stay and mortality rate), resulting in an inability to perform a meta-analysis review. Notwithstanding these limitations, this review identified research and outcomes of beta-blocker in PSH patients post-TBI. As such, it offers valuable insight into the understanding and management of TBI [41].

## 5. Conclusion

In this scoping review, we identified studies that explored the role of beta-blockers in the management of post-TBI PSH. Propranolol was the drug of choice and was shown to reduce the length of stay and mortality rate in moderate-to-severe TBI patients with PSH. No other beta-blockers in single administration were able to demonstrate the similar efficacy, probably owing to their pharmacodynamics (i.e., propranolol has lipophilic properties that allow penetration of the blood-brain barrier). More prospective studies are needed to ascertain the ideal time, dose, withdrawal, and long-term effects of beta-blocker therapy.

## Figures and Tables

**Table 1:** Summary of findings.

Articles	Study design	Participants mean or median age	TBI severity	Type of beta-blockers used	Dosage of beta-blockers	Beta-blockers were used	Non-beta-blockers drugs	Length of stay	Mortality rate
Choi et al. [8]	Narrative review	26	Moderate, severe	Propranolol	N/A	In combination	Morphine, baclofen, gabapentin, benzodiazepines, bromocriptine	N/A	N/A

Diamond et al. [21]	Case report	35.5	Severe	Labetalol	10 mg	In combination	Morphine	N/A	N/A

Feng et al. [22]	Narrative review	18.1	Severe	Propranolol	40 mg q12 h IV	In combination	Morphine, baclofen, gabapentin, benzodiazepine, bromocriptine, clonidine, dexmedetomidine, dantrolene	N/A	N/A

Godoy et al. [23]	Letter to the editor	28	Moderate, severe	Propranolol	20 mg q12 h to 20 mg q8 h IV	In combination	Morphine, fentanyl	N/A	N/A

Schroeppel et al. 2014 [24]	Case-control	42	Severe	Propranolol	85 mg IV	Alone	N/A	44	3

Van der jagt and miranda. [25]	Review	55	Moderate, severe	Atenolol	10 mg q6 h IV followed 100 mg q24 h orally	Alone	N/A	N/A	5.1

Garg et al. [26]	Case report	26	Severe	Propranolol	10 mg q12 h to 10 mg q8 h IV	Alone	N/A	N/A	N/A

Monteiro et al. [27]	Case report	27	Severe	Propranolol	10 mg q8 h	Alone	N/A	N/A	N/A

Zheng et al. [9]	Narrative review	30	Moderate, severe	Propranolol, labetalol, metoprolol	20–60 mg q4-6 hr orally	In combination	*α*2-Agonists, gabapentin, baclofen, bromocriptine, and long-acting benzodiazepines	N/A	N/A

Ammar and Hussein [11]	Randomized, double-blinded, placebo-controlled trial	55	Moderate	Propranolol	1 mg q6 hr IV	Alone	N/A	N/A	N/A

## Appendix

### A.1. Search Terms

- Paroxysmal sympathetic storm, paroxysmal sympathetic surge, diencephalic seizure, diencephalic storm, paroxysmal autonomic instability with dystonia, acute hypothalamic instability, dysautonomia, sympathetic storming, brainstem attacks, and autonomic dysregulation
- Beta-adrenergic blockers, adrenergic beta antagonists, *β*-adrenergic blockers, and *β*-blockers
- Traumatic brain injury, head injury, and craniocerebral trauma

### A.2. Search Strings

#### A.2.1. PubMed

(“Autonomic Nervous System Diseases”[MeSH Terms] OR “Paroxysmal sympathetic storm”[Text Word] OR “diencephalic seizure”[Text Word] OR “paroxysmal autonomic instability with dystonia”[Text Word] OR “acute hypothalamic instability”[Text Word]) AND (“adrenergic beta antagonists”[MeSH Terms] OR “beta-adrenergic blockers”[Text Word] OR “adrenergic beta antagonist”[Text Word] OR “beta-adrenergic blockers”[Text Word] OR “beta-blockers”[Text Word]) AND (“craniocerebral trauma”[MeSH Terms] OR (“craniocerebral”[All Fields] AND “trauma”[All Fields]) OR “craniocerebral trauma”[All Fields] OR (“head”[All Fields] AND “injury”[All Fields]) OR “head injury”[All Fields]).
